# Supplementary material for: Clinical and Pathological Diagnosis of Hereditary Gastrointestinal Polyposis in Jack Russell Terriers
Source: Vet Sci. 2022 Oct 8;9(10):551. doi: 10.3390/vetsci9100551 (PMC9612179; doi:10.3390/vetsci9100551)
Supplement: Supplementary file 1 [file vetsci-09-00551-s001.zip › vetsci-1886763-SI.pdf]

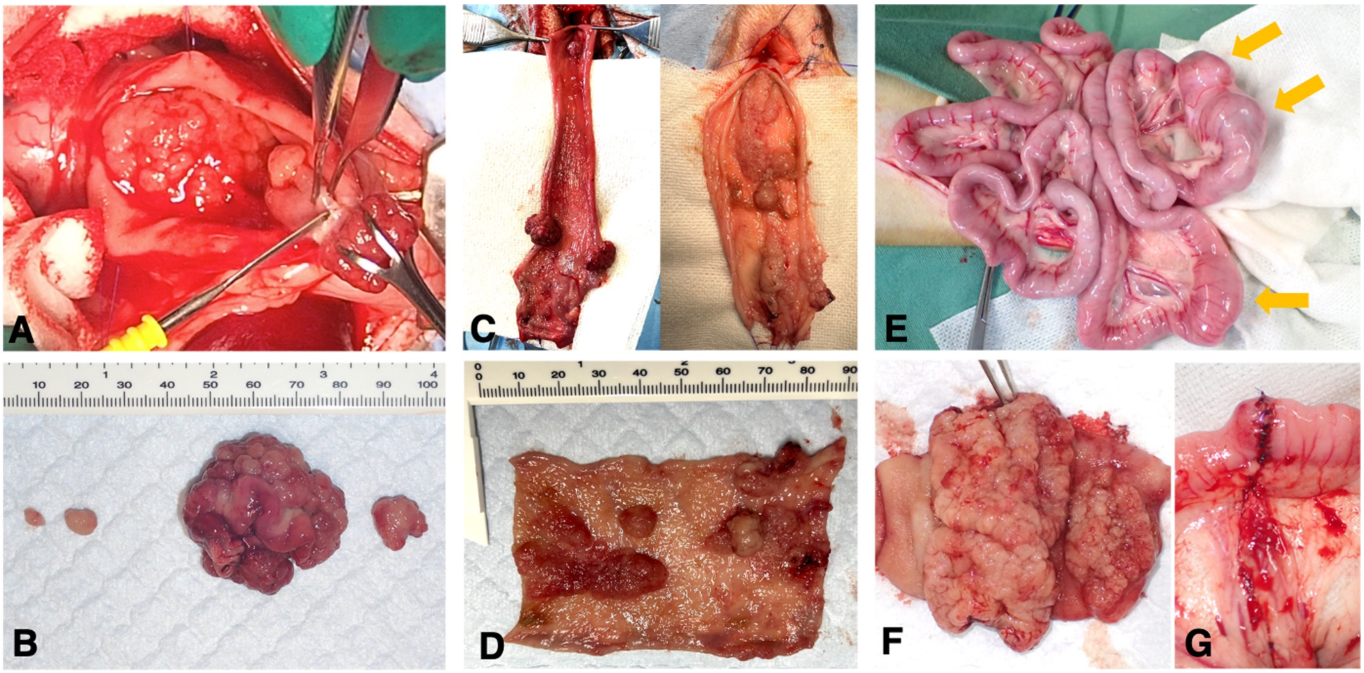

Figure S1. Various operative procedures applied to resections of gastrointestinal tumors in Jack Russell Terriers with hereditary gastrointestinal polyposis. (A, B) Submucosal resection of gastric tumors (A) and macroscopic appearance of the resected tumors (B). The mass is being lifted and resected at the submucosal layer with an electrocautery scalpel. (C, D) Colorectal mucosal pull-through procedure. Eight tumors of various sizes on the colorectal mucosa were resected by mucosal pull-through (D). (E-G) Macroscopic appearances of the ileojejunal tumors. Perioperatively, three tumors (yellow arrows) were observed in the small intestine (E). Appearance of the tumors in the longitudinally incised intestinal tract removed by end-to-end anastomosis (F). Photograph of end-to-end anastomosis after mass resection (G).

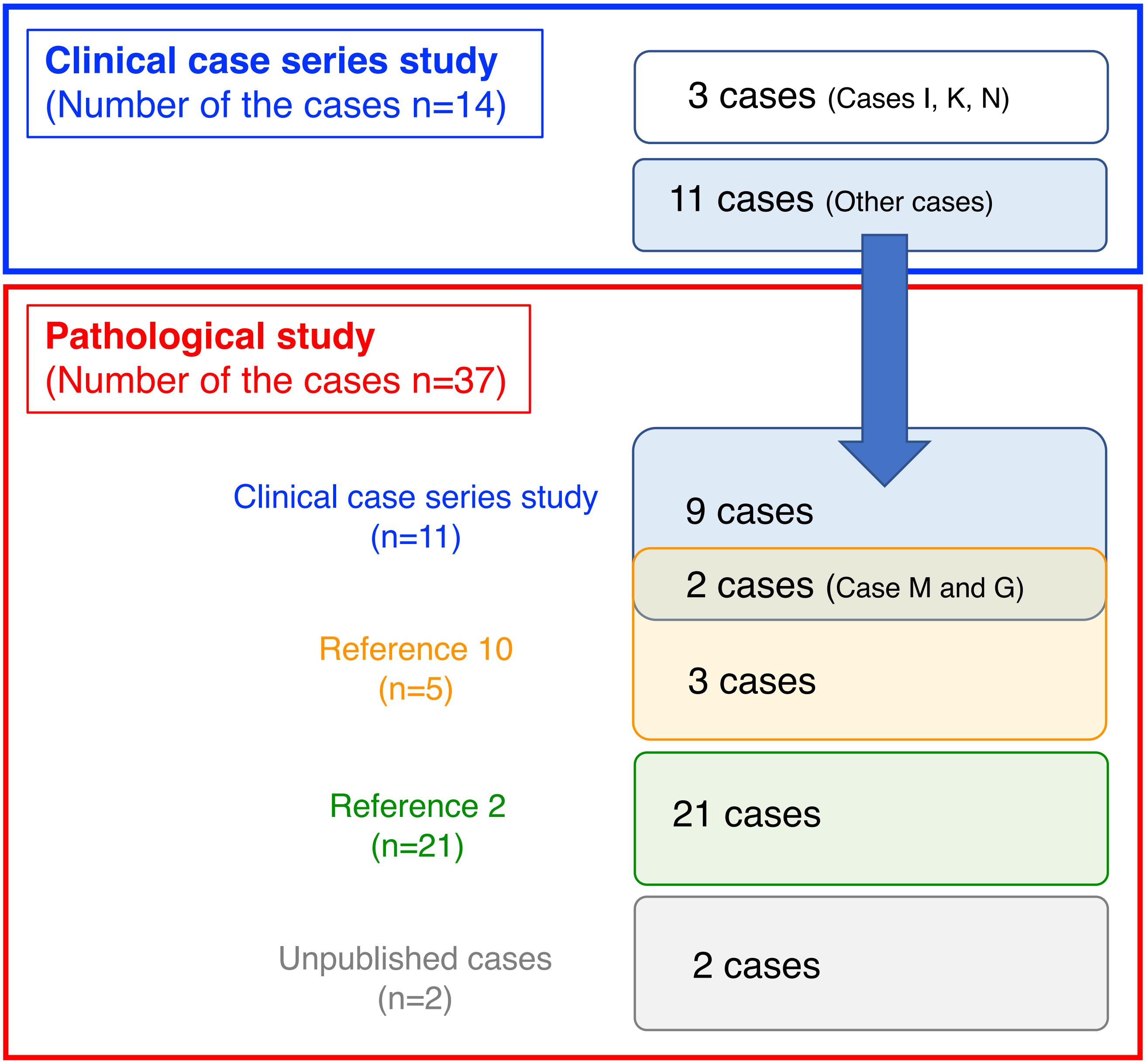

Figure S2. Schematic overview of the samples examined in the clinical and pathological studies. See also Supplementary Table 2.

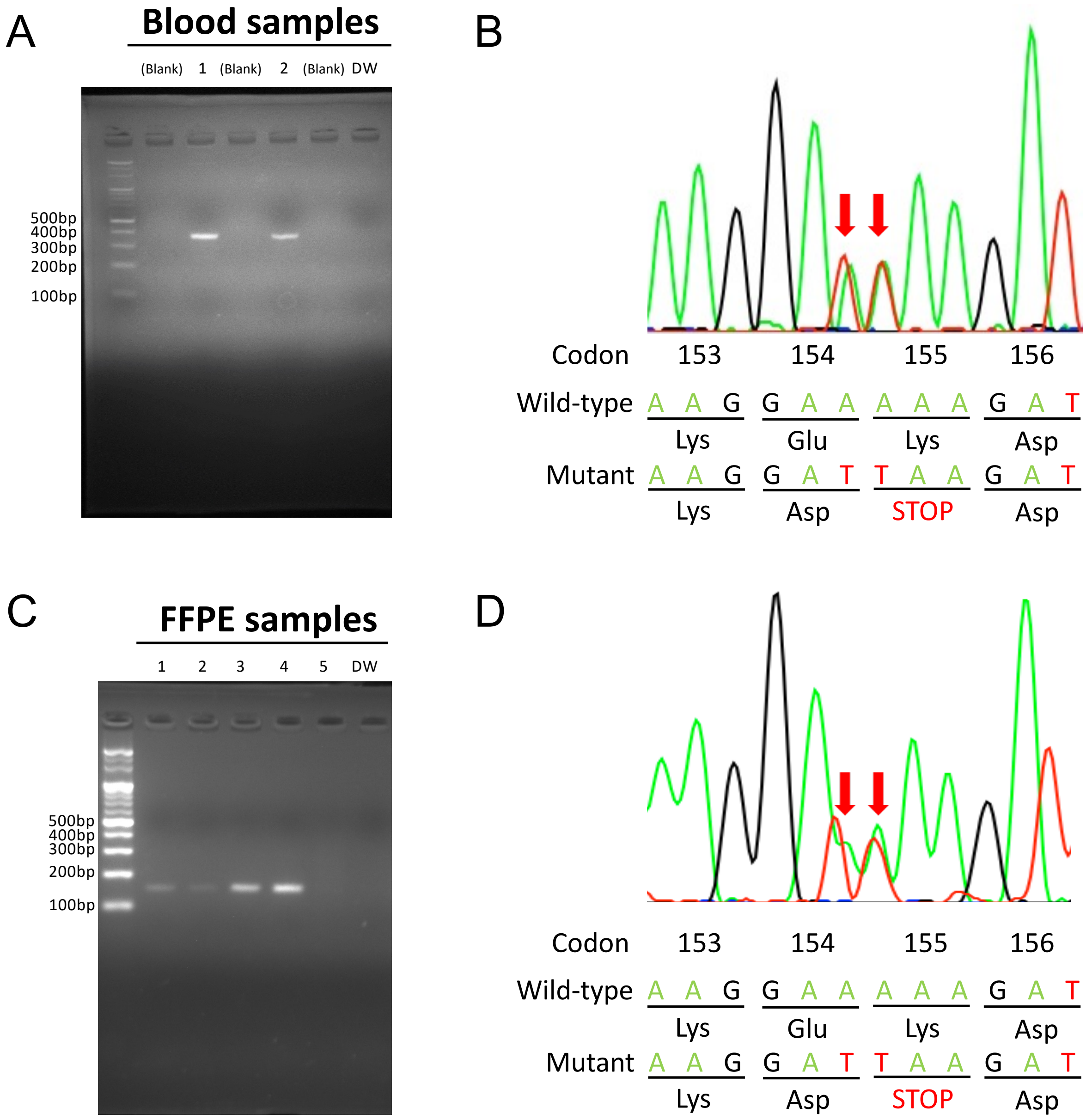

Figure S3. Genotyping assays for the hereditary gastrointestinal polyposis-associated germline *APC* variant using PCR-direct sequencing. Representative images of agarose gel electrophoresis of PCR products amplified from blood and formalin-fixed paraffin-embedded (FFPE) samples (A and C) and DNA sequencing electropherograms of codons 153-156 in the *APC* gene (B and D). (A) Cases L and N in the clinical case series study. (B) Case L. (C) Cases JRT20, 4, 10 and 1 in the pathological study (lanes 1, 2, 3 and 4). (D) Case JRT10. Red arrows indicate 2-bp substitution at codon 154-155 (c.[462_463delinsTT]).

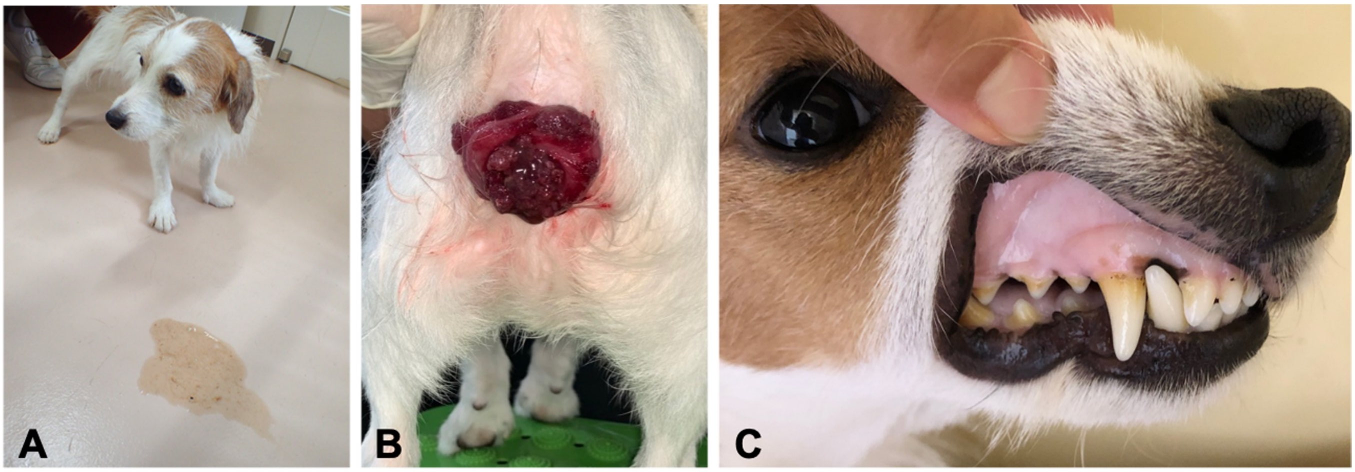

Figure S4. Common clinical signs of hereditary gastrointestinal polyposis in Jack Russell Terriers. (A) Vomiting of gastric fluid containing blood. (B) Rectal prolapse with profuse bleeding caused by tenesmus due to a rectal tumor. (C) Mucosal pallor due to anemia.

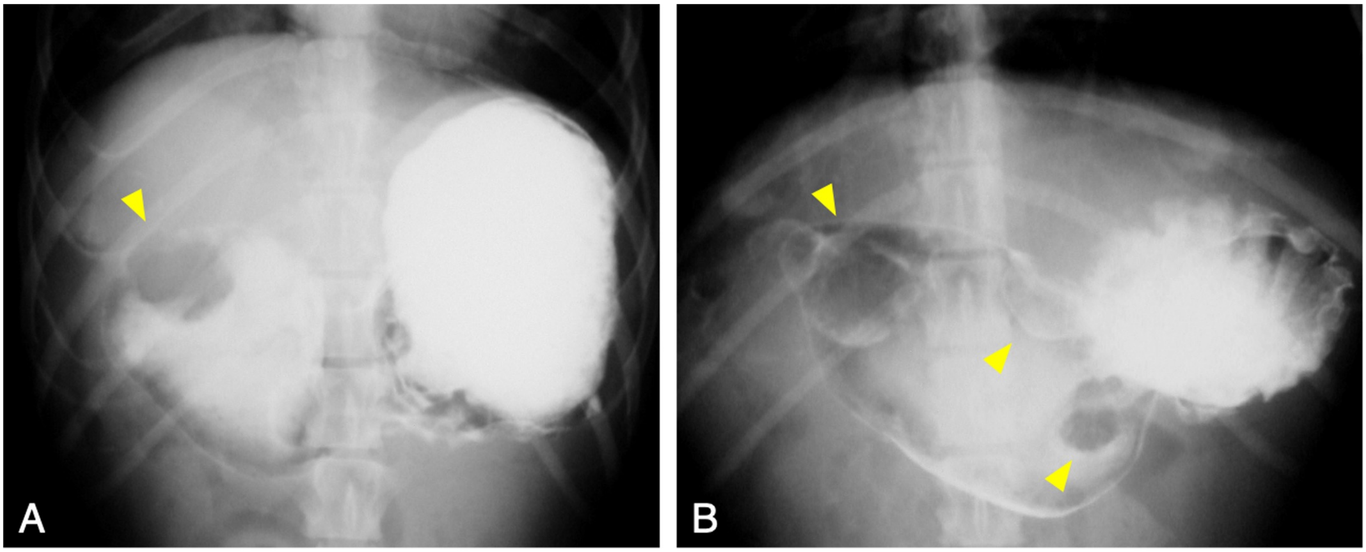

Figure S5. Representative X-ray images of gastric tumors in Jack Russell Terriers with hereditary gastrointestinal polyposis. Contrast radiographs taken immediately after barium intake (A) and one hour later (B). (A) Barium contrast radiograph reveals a mass in a pyloric area as a filling defect (yellow arrowhead). (B) On a radiograph taken one hour after barium intake, three polypoid masses (yellow arrowheads) can be visualized in the stomach due to barium adherence.

Table S1. Blood test results of Jack Russell Terriers with hereditary gastrointestinal polyposis at initial diagnosis .

Variable	RBC	Ht	WBC	Platelets	TP	ALB	GOT	GPT	T-bil	GLU	BUN	Cre	Na	K	Cl
Unit	($\times 10^4/\mu l$)	(%)	(/μl)	($\times 10^4/\mu l$)	(g/dl)	(g/dl)	(U/l)	(U/l)	(mg/dl)	(mg/dl)	(mg/dl)	(mg/dl)	(mEq/l)	(mEq/l)	(mEq/l)
Refarence range	550-850	37-55	6000-17000	20-50	5.0-7.2	2.6-4.0	17-44	17-78	0-0.5	75-128	9.2-29.2	0.4-1.4	141-152	3.8-5.0	102-117
Case No.															
A	722	48	8200	(-)	5.6	2.4	35	26	-	115	15.1	0.70	149	4.2	112
B	563	37	34000	37.2	6.4	2.5	24	18	-	141	18.4	0.62	145	3.8	106
C	769	53	14900	40	6.4	3	32	46	0.1	121	15.9	0.75	150	4.1	119
D	475	34	21600	30	5.2	2.9	35	130	0.1	125	28.1	1.12	141	3.1	97
E	256	21	33500	37.2	4.2	1.7	19	17	-	117	14.8	0.30	147	4.2	116
F	775	47	17200	30	7.2	2.6	24	61	0.1	102	10.9	1.04	(-)	(-)	(-)
G	680	45	16000	36.1	6.1	3	26	36	0.1	149	18.5	0.70	(-)	(-)	(-)
H	802	54.8	25700	25.1	5.9	2.9	30	28	-	104	8.1	0.65	148	4.5	113
I	525	21	41500	68.6	ND	ND	ND	ND	ND	ND	ND	ND	ND	ND	ND
J	635	33	35300	32	5.7	3	-	50	-	125	8.9	0.51	150	3.5	111
K	ND	ND	ND	ND	ND	ND	ND	ND	ND	ND	ND	ND	ND	ND	ND
L	689	50	19900	68	-	2.3	40	51	0.2	114	13.7	0.87	(-)	(-)	(-)
M	514	37	10400	49.5	7.2	3.6	37	82	0.1	107	21.5	0.60	(-)	(-)	(-)
N	251	18	43900	53.4	-	2	143	667	4	111	9.5	0.41	(-)	(-)	(-)
mean ± s.d.	589 ± 182	38.4 ± 12.6	24777 ± 11781	42.3 ± 14.5	6.0 ± 0.9	2.7 ± 0.5	40 ± 35	101 ± 181	0.67 ± 1.47	119 ± 14	15.2 ± 5.8	0.69 ± 0.24	147 ± 3	3.9 ± 0.5	110 ± 7
	(n=13)	(n=13)	(n=13)	(n=12)	(n=10)	(n=12)	(n=11)	(n=12)	(n=7)	(n=12)	(n=12)	(n=12)	(n=7)	(n=7)	(n=7)

ND, No available data

(-), Not examined

Table S2. Histopathological diagnosis of gastrointestinal lesions in Jack Russell Terriers

Case No.	Tumor No.	Age at first diagnosis	Age at biopsy or surgery	Sex	Method of excision		Location of GI epithelial tumor		Histopathological diagnosis[1]	Depth of invasion (T status)[2, 3,*]	Reference
JRT-01	01	10 y 5 m	10 y 5 m	M	E		Stomach	Corpus	Papillary adenoma	-	Ref. 2
JRT-02	01	12 y 4 m	12 y 4 m	M (castrated)	S	(resected by hand)	Large intestine	Rectum	Papillary adenocarcinoma	-	Ref. 2
JRT-03	01	6 y	6 y	M (castrated)	S		Large intestine	Rectum	Papillary adenocarcinoma	pTis	Ref. 2
JRT-04	01	10 y 5 m	10 y 5 m	M (castrated)	S		Large intestine	Rectum	Papillary adenocarcinoma	pTis	Ref. 2
JRT-05	01	11 y 8 m	11 y 8 m	F (spayed)	S		Large intestine	Rectum	Papillary adenocarcinoma	pTis	Ref. 2
JRT-06	01	12 y 1 m	12 y 3 m	M (castrated)	E	(polytectomy)	Stomach	Antrum	Tubulopapillary adenoma	-	Ref. 2
	02		12 y 3 m	M (castrated)	E	(polytectomy)	Stomach	Antrum	Tubulopapillary adenoma	-	
JRT-07	01	5 y 3 m	5 y 3 m	M	S		Large intestine	Rectum	Papillary adenocarcinoma	pTis	Ref. 2
	02		5 y 3 m		S		Large intestine	Rectum	Papillary adenocarcinoma	pTis	
	03		7 y 3 m		S		Large intestine	Rectum	Papillary adenocarcinoma	pTis	
JRT-08	01	7 y 10 m	7 y 10 m	M	E		Stomach	Cardia	Tubular adenoma	-	Ref. 2
	02		7 y 10 m		E		Stomach	Antrum	Tubular adenoma	-	
	03		7 y 10 m		E		Large intestine	Colon	Papillary adenocarcinoma	-	
JRT-09	01	9 y 9 m	13 y 2 m	M (castrated)	S	(pull-through)	Large intestine	Rectum	Papillary adenocarcinoma	pTis	Ref. 2
JRT-10	01	7 y 3 m	8 y 11 m	M (castrated)	S		Large intestine	Rectum	Papillary adenocarcinoma	pTis	Ref. 2
	02		8 y 11 m		S		Large intestine	Rectum	Papillary adenocarcinoma	pTis	
	03		8 y 11 m		S		Large intestine	Rectum	Papillary adenocarcinoma	pTis	
	04		8 y 11 m		S		Large intestine	Rectum	Papillary adenocarcinoma	pTis	
	05		8 y 11 m		S		Large intestine	Rectum	Adenoma	-	
JRT-11	01		12 y 5 m	M (castrated)	S	(resected by laser)	Large intestine	Rectum	Papillary adenocarcinoma	pTis	Ref. 2
JRT-12	01	4 y 1 m	7 y 8 m	F (spayed)	S	(resected by hand)	Large intestine	Rectum	Papillary adenocarcinoma	unevaluable	Ref. 2
	02		7 y 9 m		S		Stomach	Antrum	Papillary adenocarcinoma	pTis	
	03		9 y 2 m		E		Stomach	Antrum	Tubulopapillary adenoma	-	
	04		9 y 8 m		E	(polytectomy)	Stomach	Antrum	Tubulopapillary adenoma	-	
	05		13 y 3 m		S	(pull-through)	Large intestine	Rectum	Papillary adenocarcinoma	pTis	
JRT-13	01	8 y 0 m	8 y 0 m	M	E		Stomach	Corpus	Papillary adenocarcinoma	-	Ref. 2
JRT-14	01	5 y 2 m	8 y 9 m	F	E		Stomach	Cardia	Tubulopapillary adenoma	-	Ref. 2
	02		8 y 9 m		E		Large intestine	Rectum	Papillary adenocarcinoma	-	
JRT-15	01	11 y 4 m	11 y 4 m	F (spayed)	S	(partial resection)	Stomach	Antrum	Tubular adenocarcinoma	(pT2) [4]	Clinical study (Case M), Ref. 10
JRT-16	01	6 y 2 m	9 y 1 m	F	S	(Billroth I)	Stomach	Antrum	Tublopapillary adenocarcinoma	pTis	Ref. 2
JRT-17	01	6 y 6 m	8 y 6 m	F (spayed)	S	(Billroth I)	Stomach	Antrum	Tubular adenocarcinoma	pT1a	Ref. 2
	02		8 y 6 m		S	(Billroth I)	Stomach	Corpus	Tubulopapillary adenoma	-	
JRT-18	01	10 y 3 m	10 y 3 m	F (spayed)	E		Stomach	Antrum	Hyperplastic polyp	-	Ref. 2
	02		10 y 3 m		E		Stomach	Antrum	Hyperplastic polyp	-	
	03		10 y 3 m		E		Stomach	Antrum	Tubulopapillary adenoma	-	
	04		10 y 3 m		E		Large intestine	Rectum	Papillary adenocarcinoma	-	
JRT-19	01	9 y 7 m	9 y 7 m	F (spayed)	S		Stomach	Antrum	Tublopapillary adenocarcinoma	pTis	-
JRT-20	01	7 y 8 m	7 y 8 m	F (spayed)	S		Stomach	Antrum	Tublopapillary adenoma	pTis	Ref. 2
	02		13 y 10 m		S		Stomach	Cardia	Tubular adenocarcinoma	pT1a	
	03		13 y 10 m		S		Stomach	Antrum	Tublopapillary adenoma	pTis	
JRT-21	01	7 y 6 m	7 y 6 m	M (castrated)	E		Large intestine	Rectum	Papillary adenocarcinoma	-	-
JRT-22	01	2 y 3 m	2 y 4 m	M	S		Stomach	Antrum	Tublopapillary adenoma	-	Ref. 2
	02				S		Stomach	Antrum	Tublopapillary adenoma	-	
	03				S		Stomach	Antrum	Papillary adenoma	-	
	04				S		Stomach	Antrum	Tublopapillary adenoma	-	
JRT-23	01	7 y 4 m	7 y 4 m	F	E		Stomach	Antrum	Tubulopapillary adenocarcinoma	-	Ref. 10
	02		7 y 4 m		E		Stomach	Antrum	Tubulopapillary adenocarcinoma	-	
JRT-24	01	6 y 1 m	6 y 1 m	M (castrated)	E		Large intestine	Rectum	Papillary adenocarcinoma	-	Ref. 10
JRT-25	01	8 y 2 m	8 y 2 m	F (spayed)	S	(local excision)	Stomach	Cardia	Tubular adenocarcinoma	pTis	Clinical study (Case L)
	02		8 y 2 m		S	(local excision)	Stomach	Cardia	Tubular adenocarcinoma	pTis	
	03		8 y 2 m		S	(local excision)	Stomach	Antrum	Tubulopapillary adenocarcinoma	pTis	
JRT-26	01	6 y 6 m	6 y 6 m	M (castrated)	E		Stomach	Antrum	Tubulopapillary adenocarcinoma	-	Clinical study (Case G), Ref. 10
	02		6 y 8 m		S	(pull-through)	Large intestine	Rectum	Papillary adenocarcinoma	pTis	
	03		6 y 8 m		S	(pull-through)	Large intestine	Rectum	Papillary adenocarcinoma	pTis	
	04		6 y 8 m		S	(pull-through)	Large intestine	Rectum	Papillary adenocarcinoma	pTis	
	05		6 y 8 m		S	(pull-through)	Large intestine	Rectum	Papillary adenocarcinoma	pTis	
	06		6 y 8 m		S	(pull-through)	Large intestine	Rectum	Papillary adenocarcinoma	pTis	
JRT-27	01		6 y 9 m		E		Small intestine	Duodenum	Papillary adenocarcinoma	-	Clinical study (Case H)
	02		6 y 9 m		E		Large intestine	Colon	Papillary adenocarcinoma	-	
JRT-28	01	4 y 7 m	5 y 4 m		E		Stomach	Not specifed	Tubulopapillary adenoma	-	Clinical study (Case E)
	02		5 y 4 m		E		Large intestine	Colon	Papillary adenocarcinoma	-	
	03		5 y 4 m		S	(pull-through)	Large intestine	Rectum	Papillary adenocarcinoma	pTis	
JRT-29	01	5y	5y	M (castrated)	E	(polytectomy)	Stomach	Antrum	Tubulopapillary adenocarcinoma	-	Ref. 2
	02		5y		S	(resected by hand)	Large intestine	Rectum	Papillary adenocarcinoma	-	
JRT-30	01	3y	3y	F	S		Stomach	Antrum	Tubulopapillary adenocarcinoma	pTis	Ref. 2
JRT-31	01	6 y 2 m	6 y 2 m	F (spayed)	E		Stomach	Antrum	Tubulopapillary adenocarcinoma	-	Ref. 10
JRT-32	01	4 y 10 m	4 y 10 m	F (spayed)	E		Large intestine	Colon	Papillary adenocarcinoma	-	Clinical study (Case F)
	02		4 y 11 m		E		Stomach	Not specifed	Tubulopapillary adenoma	-	
	03		5 y 0 m		S		Large intestine	Rectum	Papillary adenocarcinoma	pTis	
	04		6 y 0 m		S	(end to end anastomosis)	Small intestine	Jejunum	Papillary adenocarcinoma	pTis	
	05		6 y 0 m		S	(end to end anastomosis)	Small intestine	Jejunum	Papillary adenocarcinoma	pTis	
	06		6 y 0 m		S	(local excision)	Stomach	Antrum	Tubulopapillary adenocarcinoma	pTis	
	07		6 y 0 m		S	(local excision)	Stomach	Antrum	Tubulopapillary adenocarcinoma	pTis	
	08		6 y 1 m		S	(pull-through)	Large intestine	Rectum	Papillary adenocarcinoma	pTis	
	09		6 y 1 m		S	(pull-through)	Large intestine	Rectum	Papillary adenocarcinoma	pTis	
	10		6 y 1 m		S	(pull-through)	Large intestine	Rectum	Papillary adenocarcinoma	pTis	
	11		6 y 1 m		S	(pull-through)	Large intestine	Rectum	Papillary adenocarcinoma	pTis	
	12		6 y 1 m		S	(pull-through)	Large intestine	Rectum	Papillary adenocarcinoma	pTis	
	13		6 y 1 m		S	(pull-through)	Large intestine	Rectum	Papillary adenocarcinoma	pTis	
	14		6 y 1 m		S	(pull-through)	Large intestine	Rectum	Papillary adenocarcinoma	pTis	
	15		6 y 1 m		S	(pull-through)	Large intestine	Rectum	Papillary adenocarcinoma	pTis	
	16		6 y 1 m		S	(pull-through)	Large intestine	Rectum	Papillary adenocarcinoma	pTis	
	17		6 y 1 m		S	(pull-through)	Large intestine	Rectum	Papillary adenocarcinoma	unevaluable	

	18		6 y 1 m		S (pull-through)	Large intestine	Rectum	Papillary adenocarcinoma	unevaluable	
	19		6 y 1 m		S (pull-through)	Large intestine	Rectum	Papillary adenocarcinoma	unevaluable	
	20		6 y 1 m		S (pull-through)	Large intestine	Rectum	Papillary adenocarcinoma	unevaluable	
	21		6 y 9 m		S (local excision)	Stomach	Antrum	Tubulopapillary adenocarcinoma	pTis	
	22		6 y 9 m		S (local excision)	Stomach	Antrum	Tubulopapillary adenocarcinoma	pTis	
	23		6 y 9 m		S (local excision)	Stomach	Antrum	Tubulopapillary adenocarcinoma	pTis	
	24		6 y 9 m		S (local excision)	Stomach	Antrum	Tubulopapillary adenocarcinoma	pTis	
	25		6 y 9 m		S (local excision)	Stomach	Antrum	Tubulopapillary adenocarcinoma	pTis	
	26		6 y 9 m		S (local excision)	Stomach	Antrum	Tubulopapillary adenocarcinoma	unevaluable	
	27		6 y 9 m		S (local excision)	Stomach	Antrum	Tubulopapillary adenoma	-	
JRT-33	01	7 y 4 m	7 y 5 m	F	S (pull-through)	Large intestine	Rectum	Papillary adenocarcinoma	pTis	Clinical study (Case J)
	02		7 y 5 m		S (pull-through)	Large intestine	Rectum	Papillary adenocarcinoma	pTis	
	03		7 y 5 m		S (pull-through)	Large intestine	Rectum	Papillary adenocarcinoma	pTis	
	04		7 y 5 m		S (pull-through)	Large intestine	Rectum	Papillary adenocarcinoma	pTis	
	05		7 y 5 m		S (pull-through)	Large intestine	Rectum	Papillary adenocarcinoma	pTis	
	06		7 y 5 m		S (pull-through)	Large intestine	Rectum	Papillary adenocarcinoma	pTis	
	07		7 y 5 m		S (pull-through)	Large intestine	Rectum	Papillary adenocarcinoma	pTis	
	08		7 y 5 m		S (pull-through)	Large intestine	Rectum	Papillary adenocarcinoma	pTis	
JRT-34	01	3 y 4 m	3 y 6 m	F	E	Small intestine	Duodenum	Papillary adenocarcinoma	-	Clinical study (Case A)
	02		4 y 7 m		S	Small intestine	Jejunum/Ilenum	Papillary adenocarcinoma	pT2	
	03		4 y 7 m		S	Small intestine	Jejunum/Ilenum	Papillary adenocarcinoma	pT1a	
	04		4 y 7 m		S	Small intestine	Jejunum/Ilenum	Papillary adenocarcinoma	pT1a	
	05		5 y 2 m		E	Stomach	Antrum	Tubulopapillary adenocarcinoma	-	
JRT-35	01	3 y 3 m	3 y 3 m	M (castrated)	E	Stomach	Not specifed	Hyperplastic polyp	-	Clinical study (Case B)
	02		3 y 3 m		E	Small intestine	Duodenum	Papillary adenocarcinoma	-	
	03		3 y 3 m		E	Large intestine	Colon	Papillary adenocarcinoma	-	
	04		3 y 3 m		E	Large intestine	Rectum	Papillary adenocarcinoma	-	
	05		4 y 2 m		S (pull-through)	Large intestine	Colon	Papillary adenocarcinoma	pTis	
	06		4 y 2 m		S (pull-through)	Large intestine	Colon	Papillary adenocarcinoma	pTis	
	07		4 y 2 m		S (pull-through)	Large intestine	Colon	Papillary adenocarcinoma	pTis	
	08		4 y 2 m		S (pull-through)	Large intestine	Colon	Papillary adenocarcinoma	pTis	
	09		4 y 2 m		S (pull-through)	Large intestine	Colon	Papillary adenocarcinoma	pTis	
	10		4 y 2 m		S (pull-through)	Large intestine	Rectum	Acinar adenocarcinoma	pTis	
	11		4 y 2 m		S (pull-through)	Large intestine	Rectum	Papillary adenocarcinoma	pTis	
	12		4 y 2 m		S (pull-through)	Large intestine	Rectum	Papillary adenocarcinoma	pTis	
	13		4 y 2 m		S (pull-through)	Large intestine	Rectum	Papillary adenocarcinoma	pTis	
	14		4 y 2 m		S (pull-through)	Large intestine	Rectum	Papillary adenocarcinoma	pTis	
JRT-36	01	3 y 11 m	3 y 11 m	F	S (pull-through)	Large intestine	Rectum	Papillary adenocarcinoma	pT1	Clinical study (Case C)
	02		3 y 11 m		S (pull-through)	Large intestine	Rectum	Papillary adenocarcinoma	pTis	
	03		3 y 11 m		S (pull-through)	Large intestine	Rectum	Papillary adenocarcinoma	pTis	
	04		3 y 11 m		S (pull-through)	Large intestine	Rectum	Papillary adenocarcinoma	pTis	
JRT-37	01	4 y 3 m	4 y 3 m	F	S (local excision)	Stomach	Corpus	Tubulopapillary adenocarcinoma	pTis	Clinical study (Case D)
	02		4 y 3 m		S (local excision)	Stomach	Corpus	Tubulopapillary adenocarcinoma	pTis	
	03		4 y 3 m		S (local excision)	Stomach	Corpus	Tubulopapillary adenocarcinoma	unevaluable	
	04		4 y 3 m		S (local excision)	Stomach	Corpus	Tubulopapillary adenocarcinoma	unevaluable	
	05		4 y 3 m		S (local excision)	Stomach	Antrum	Tubulopapillary adenocarcinoma	pTis	
	06		4 y 3 m		S (local excision)	Stomach	Antrum	Tubulopapillary adenocarcinoma	pTis	

y: years, m: months, M: Male, F: Female, E: Endoscopic biopsy, S: Surgical resection

[1] Histological Classification of Tumours of the Alimentary System of Domestic Animals (WHO International Classification of Tumors of Domestic Animal Series) (Ref. 2)

[2] TNM classification of malignant tumors (Ref. 13)

[3] AJCC cancer staging manual (Ref. 12)

[4] It was not possible to examine the tissues deeper than muscularis propria due to partial resection.

*T (tumor) status, the depth of invasion, are evaluated according to the following criteria.

Stomach:
Tis: Intraepithelial tumor without invasion of the lamina propria (carcinoma in situ), T1a: Tumor invades lamina propria or muscularis mucosae, T1b: Tumor invades submucosa, T2 Tumor invades muscularis propria, T3: Tumor invades subserosa, T4a: Tumor perforates serosa (visceral peritoneum), T4b: Tumor invades adjacent structures.

Small intestine:
Tis: Carcinoma in situ, T1a: Tumor invades lamina propria or muscularis mucosae, T1b: Tumor invades submucosa, T2: Tumor invades muscularis propria, T3: Tumor invades subserosa or nonperitonealized perimuscular tissue (mesentery or retroperitoneum) with extension 2 cm or less, T4: Tumor perforates visceral peritoneum or directly invades other organs or structure (includes other loops of small intestine, mesentery, or retroperitoneum more than 2 cm and abdominal wall by way of serosa; for duodenum only. invasion of pancreas)

Large intestine:
Tis: Intraepithelial or invasion of lamina propria (carcinoma in situ), T1: Tumor invades submucosa, T2: Tumor invades muscularis propria, T3: Tumor invades subserosa or into non-peritonealized pericolic or perirectal tissue, T4a: Tumor perforates visceral peritoneum, T4b: Tumor directly invades other organs or structure.

Table S3. Case information and histopathological findings of sporadic gastrointestinal adenocarcinomas of multiple dog breeds other than JRTs

Case No.	Age at diagnosis	Sex	Dog breed	Location of tumor	Histopathological diagnosis[1]	Depth of invasion (T status)[2, 3,*]
SC-01	10 y 6 m	Male	Toy Poodle	Stomach	Tubulopapillary adenocarcinoma	pTis
SC-02	9 y	Female	Border Collie	Stomach	Signet-ring cell carcinoma	pT3
SC-03	6 y 3 m	Female (spayed)	Toy Poodle	Stomach	Tubulopapillary adenocarcinoma	pTis
SC-04	12 y 7 m	Female (spayed)	Mixed-breed	Stomach	Signet-ring cell carcinoma	pT3
SC-05	N.D.	Male (castrated)	French Bulldog	Stomach	Tubulopapillary adenocarcinoma	pTis
SC-06	7 y 11 m	Male (castrated)	Miniature Dachshund	Stomach	Signet-ring cell carcinoma	pT4a
SC-07	7 y 8 m	Female	Toy Poodle	Stomach	Tubulopapillary adenocarcinoma	pTis
SC-08	9 y 2 m	Female (spayed)	Mixed-breed (Japanese dog)	Small intestine	Signet-ring cell carcinoma	pT3
SC-09	12 y 3 m	Male	Miniature Dachshund	Small intestine (Ileum)	Papillary adenocarcinoma	pT3
SC-10	14 y	Male	Miniature Dachshund	Small intestine	Acinar adenocarcinoma	pT2
SC-11	7 y 10 m	Female	Miniature Pinscher	Small intestine (Jejunum-Ileum)	Acinar adenocarcinoma	pT2
SC-12	13 y	Female (spayed)	Toy Poodle	Small intestine	Acinar adenocarcinoma	pT3
SC-13	13 y 9 m	Male	Miniature Dachshund	Small intestine (Ileum)	Papillary adenocarcinoma	pTis
SC-14	13 y	Female	Toy Poodle	Small intestine	Mucinous adenocarcinoma	pT3
SC-15	14 y	Female (spayed)	Shih Tzu	Small intestine	Acinar adenocarcinoma	pT3
SC-16	12 y 5 m	Female (spayed)	Miniature Schnauzer	Small intestine (Duodenum-Jejunum)	Mucinous adenocarcinoma	pT3
SC-17	7 y	Male (castrated)	Shiba Inu	Small intestine	Acinar adenocarcinoma	pT3
SC-18-1	11 y 3 m	Female (spayed)	American Cocker Spaniel	Small intestine (Ileum)	Papillary adenocarcinoma	pT2
SC-18-2				Small intestine (Ileum)	Papillary adenocarcinoma	pT2
SC-19	13 y 1 m	Male	Miniature Dachshund	Small intestine (Ileum)	Papillary adenocarcinoma	pT2
SC-20	9 y 6 m	Female	Chihuahua	Small intestine (Ileum)	Papillary adenocarcinoma	pT2
SC-21	8 y 6 m	Female (spayed)	Miniature Dachshund	Large intestine (Rectum)	Acinar adenocarcinoma	pTis
SC-22	9 y 5 m	Male (castrated)	Toy Poodle	Large intestine (Rectum)	Papillary adenocarcinoma	pTis
SC-23	10 y 3 m	Male (castrated)	Mixed-breed	Large intestine (Rectum)	Papillary adenocarcinoma	pTis
SC-24	8 y 8 m	Female	Toy Poodle	Large intestine (Cecum)	Papillary adenocarcinoma	pT3
SC-25-1	9 y 5 m	Male (castrated)	Welsh Corgi	Large intestine (Colon)	Papillary adenocarcinoma	pTis
SC-25-2				Large intestine (Colon)	Papillary adenocarcinoma	pT2
SC-26-1	9 y 5 m	Male	Shiba Inu	Large intestine (Rectum)	Papillary adenocarcinoma	pTis
SC-26-2				Large intestine (Rectum)	Papillary adenocarcinoma	pTis
SC-26-3				Large intestine (Rectum)	Acinar adenocarcinoma	pTis
SC-27	3 y 0 m	Male (castrated)	Shetland Sheepdog	Large intestine (Rectum)	Papillary adenocarcinoma	pTis
SC-28	6 y 9 m	Male	Mixed-breed	Large intestine (Rectum)	Papillary adenocarcinoma	pTis
SC-29	1 y 0 m	Female (spayed)	Labrador Retriever	Large intestine (Rectum)	Papillary adenocarcinoma	pTis
SC-30	7 y 8 m	Male (castrated)	Toy Poodle	Large intestine (Rectum)	Papillary adenocarcinoma	pTis
SC-31	8 y 0 m	Male (castrated)	Yorkshire Terrier	Large intestine (Rectum)	Papillary adenocarcinoma	pTis
SC-32	10 y 1 m	Male (castrated)	Yorkshire Terrier	Large intestine (Rectum)	Papillary adenocarcinoma	pTis
SC-33	4 y 11 m	Male (castrated)	Shetland Sheepdog	Large intestine (Rectum)	Papillary adenocarcinoma	pTis
SC-34	10 y	Female (spayed)	Shiba Inu	Large intestine (Rectum)	Papillary adenocarcinoma	pTis
SC-35	3 y	Male	Pomeranian	Large intestine (Rectum)	Papillary adenocarcinoma	pTis
SC-36	9 y	Male (castrated)	Chihuahua	Large intestine (Rectum)	Acinar adenocarcinoma	pT1
SC-37	8 y 4 m	Male (castrated)	Standard Poodle	Large intestine (Rectum)	Undifferentiated carcinoma	pT3
SC-38	9 y 5 m	Male	Labrador Retriever	Large intestine (Rectum)	Papillary adenocarcinoma	pTis
SC-39	13 y 10 m	Male (castrated)	Toy Poodle	Large intestine (Rectum)	Papillary adenocarcinoma	pTis
SC-40	9 y 10 m	Female (spayed)	Mixed-breed	Large intestine (Rectum)	Papillary adenocarcinoma	pTis

y: years, m: months, M: Male, F: Female

[1] Histological Classification of Tumours of the Alimentary System of Domestic Animals (WHO International Classification of Tumors of Domestic Animal Series) (Ref. 2)

[2] TNM classification of malignant tumors (Ref. 13)

[3] AJCC cancer staging manual (Ref. 12)

* T (tumor) status, the depth of invasion, are evaluated according to the criteria below.

Stomach
Tis: Intraepithelial tumor without invasion of the lamina propria (carcinoma in situ), T1a: Tumor invades lamina propria or muscularis mucosae, T1b: Tumor invades submucosa, T2 Tumor invades muscularis propria, T3: Tumor invades subserosa, T4a: Tumor perforates serosa (visceral peritoneum), T4b: Tumor invades adjacent structures.

Small intestine
Tis: Carcinoma in situ, T1a: Tumor invades lamina propria or muscularis mucosae, T1b: Tumor invades submucosa, T2: Tumor invades muscularis propria, T3: Tumor invades subserosa or nonperitonealized perimuscular tissue (mesentery or retroperitoneum) with extension 2 cm or less, T4: Tumor perforates visceral peritoneum or directly invades other organs or structure.

Large intestine
Tis: Intraepithelial or invasion of lamina propria (carcinoma in situ), T1: Tumor invades submucosa, T2: Tumor invades muscularis propria, T3: Tumor invades subserosa or into non-peritonealized pericolic or perirectal tissue, T4a: Tumor perforates visceral peritoneum, T4b: Tumor directly invades other organs or structure.

Table S4. Comparison of histolpathological diagnosis between endoscopically and surgically resected samples of gastrointestinal lesions in Jack Russell Terriers with the germline *APC* variant

Case No.	Tumor No.*	Age at diagnosis	Method of excision	Location of tumor	Histopathological diagnosis	Histological type
JRT-12	02	7 y 8 m	Endoscopic biopsy	Stomach (Antrum)	No significant lesion	
		7 y 9 m	Surgically resection		Adenocarcinoma	Papillary
JRT-20	02	13 y 10 m	Endoscopic biopsy	Stomach (Cardia)	Adenoma	Papillary
		13 y 10 m	Surgically resection		Adenocarcinoma	Tubular
	03	13 y 10 m	Endoscopic biopsy	Stomach (Antrum)	Adenoma	Papillary
		13 y 10 m	Surgically resection		Adenoma	Tubulopapillary
JRT-22	01	2 y 3 m	Endoscopic biopsy	Stomach (Antrum)	Adenoma	Tubular
		2 y 4 m	Surgically resection		Adenoma	Tubulopapillary
JRT-26	02	6 y 6 m	Endoscopic biopsy	Large intestine (Rectum)	Adenocarcinoma	Papillary
		6 y 8 m	Surgically resection (pull-through)		Adenocarcinoma	Papillary
JRT-33	01	7y 4m	Endoscopic biopsy	Large intestine (Rectum)	Adenocarcinoma	Papillary
		7 y 5 m	Surgically resection (pull-through)		Adenocarcinoma	Papillary

y: years, m: months

* See Supplementary Table 1
